# Hope therapy brings hope: an empirical study of a curriculum intervention to enhance school adaptation of Chinese high school freshmen

**DOI:** 10.3389/fpsyg.2025.1555364

**Published:** 2025-04-28

**Authors:** Zuobing Deng, Rui Yang, Ning Li, Xin Wei, Yan Li

**Affiliations:** ^1^School of Teacher Development, Shaanxi Normal University, Xi'an, China; ^2^Renhuai No.6 High School, Zunyi, China; ^3^Faculty of Psychology, Beijing Normal University, Beijing, China; ^4^Bijie Education Science Research Institute, Bijie, China; ^5^Middle School, Karamay School Attached to Beijing Normal University, Karamay, China

**Keywords:** hope therapy, school adaptation, high school freshmen, mental health curriculum, mental health education

## Abstract

Adolescents often face significant school adaptation challenges during the school transition period, which may negatively affect their academic performance, emotional well-being, and long-term development. However, research on school adaptation interventions remains limited, especially in applying hope therapy within large-scale, curriculum-based models in the Chinese high school context. This study examined the empirical effects of an 8-week mental health intervention curriculum based on hope therapy in enhancing school adaptation with Chinese freshmen high school students. A pretest revealed that the natural stabilization time for freshmen school adaptation was the ninth week after enrollment. A subsequent formal intervention experiment used a pre- and post-test randomized group control design to divide 444 students into matched experimental and control groups. The experimental group participated in eight psychological intervention sessions based on hope therapy, and the control group received regular mental health lessons. Results showed that the experimental group’s hope level and school adaptation significantly increased after the intervention, not only exceeding those of the pre-intervention level, but also significantly higher than those of the control group. Cross-lagged modeling results further revealed that hope levels at earlier time points significantly predicted subsequent school adaptation, while the reverse effect was not significant. These results collectively support the validity and applicability of hope therapy in improving adolescents’ school adaptation, and provides innovative and alternative intervention perspectives and practice guidelines for high school mental health education, with a strong focus on raising youths’ hope to develop meaningful lives.

## 1 Introduction

Adolescents face various new school adaptation challenges during the transition process, such as academic pressure, peer pressure, conflict with parents, and emotional management difficulties (Zhu et al., 2021). In China, due to the prevalence of examination culture and the strictness of school management style, high school students face increasing school adaptation issues, which may lead to negative psychological states such as poor academic performance, lack of social skills, increased emotional distress and problematic behaviors (Amai, 2020), and low life satisfaction (Zeng et al., 2022), and may even result in academic burnout, depression, anxiety, and even dropout (Méndez et al., 2021). Studies have shown that school adaptation of Chinese high school students has gradually become a typical and important concern that has attracted extensive attention from researchers, schools, and families (Li and Li, 2018). In response, the Chinese Ministry of Education has issued several policies, such as the Special Action Plan for Comprehensively Strengthening and Improving Students’ Mental Health Work in the New Era (2023–2025) and the Circular of the General Office of the Ministry of Education on Strengthening the Management of Students’ Mental Health, to support the implementation of the mental health curriculum and the selection, recruitment, and training of psychologists in high schools, and to address the increasingly common and serious student mental health problems. Therefore, how to effectively tackle the increasingly prevalent school adaptation problems of Chinese high school freshmen through mental curriculum and counseling is of high research and practical value.

School serves as a primary microsystem for adolescent development, and school adaptation is a critical indicator of adolescents’ psychosocial well-being (Azpiazu et al., 2024). School adaptation refers to the students’ proactive adjustment and coping capacity when facing academic, social, and emotional challenges within school environments (Kiuru et al., 2009; Rodríguez-Fernández et al., 2016). School adaptation *is considered as a multidimensional construct that* primarily reflects students’ academic adaptation, attitudes toward school, social relationships (peer and teacher-student relationships), and behavioral adaptation (rule compliance and prosocial conduct) (Azpiazu et al., 2024; Fengjun et al., 2022). Higher levels of school adaptation correlate significantly with elevated school satisfaction and overall life well-being (Eoh et al., 2022). Effective school adaptation not only enhances academic achievement and classroom engagement but also supports emotional regulation, self-concept (Engels et al., 2019), and peer relationships (Chen et al., 2000). However, many adolescents face notable challenges in adapting to school, particularly during developmental transitions, such as entering high school. These difficulties often manifest as reduced optimism about the future, lower academic self-efficacy, and signs of maladaptation (Kiuru et al., 2020; Serna and Martínez, 2019). Importantly, school adaptation is influenced not only by academic performance but also by psychological factors and social supports, such as mental health, positive academic attitudes (Xie et al., 2022), family, community, and peer networks (Dias and Cadime, 2017). This highlights its malleability and potential for targeted interventions (Evans and Bakhtiari, 2024). Therefore, exploring systematic strategies to enhance school adaptation among high school freshmen has become a pressing issue requiring immediate attention.

Currently, intervention research addressing adolescent school adaptation remains limited, focusing primarily on enhancing adaptability, psychological resilience, and social support (Donaldson et al., 2023). For example, programs like Positive Transition (Coelho et al., 2017; Coelho et al., 2018) and Coping Power (Lochman and Wells, 2002) demonstrated effectiveness in helping middle school students develop coping skills and improve school adaptation by managing expectations, time planning, and self-assessment. Durlak et al. (2011) used meta-analysis to reveal the benefits of social–emotional learning programs, showing significant short-term positive impacts. Similarly, Stormshak et al. (2011) implemented a family-centered intervention involving parent counseling, promoting social adaptation and reduced health-risk behaviors among adolescents. These findings highlight the potential of school-based interventions in promoting adaptation. However, most programs are highly targeted, focusing on small groups of at-risk students through time-and labor-intensive approaches (Tornivuori et al., 2023), which may not be suitable for broader school populations undergoing transition-related adaptation issues. To address these limitations, curriculum-based approaches that integrated positive emotions and emotion regulation were applied to enhance adaptation in Finnish primary schools (Sourander et al., 2024). In addition, Chen C. et al. (2024) and Chen X. et al. (2024) conducted a randomized controlled trial evaluating a well-being promotion program for secondary school students, demonstrating significant improvements in well-being and adaptation. These findings underscore the potential of positive psychology-based interventions in designing scalable, school-wide curricula and in supporting students’ school adaptation.

Hope therapy, a key intervention model in positive psychology, conceptualizes hope as a dynamic cognitive-motivational system. According to Snyder’s hope theory, hope is defined as the cognitive processes employed in the pursuit of goals, facilitated by two key cognitive components: pathways thinking and agency thinking (Snyder et al., 1991). Pathways thinking refers to an individual’s ability to generate feasible routes to goal achievement, while maintaining confidence in the likelihood that the chosen path will lead to success (Snyder et al., 2002). High-hope individuals are also capable of generating alternative pathways to reach their goals if their current path is blocked. Agency thinking, on the other hand, is the motivational component of hope theory. It involves utilizing self-referential thoughts to generate the confidence and mental energy necessary to initiate and sustain the pursuit of pathways throughout the goal achievement process (Snyder et al., 2002). Both pathways and agency thinking are considered iterative and additive elements that are crucial for successful goal attainment. Research has shown that individuals with higher levels of hope tend to achieve greater success across various domains, such as academics and sports, indicating that hope plays a critical role in goal achievement and perseverance (Corrigan and Schutte, 2023). Hope therapy focuses on strengthening hope through three core components—goal clarification, pathway development, and motivational enhancement—which collectively promote mental health and behavioral improvements (Lochman and Wells, 2002; Snyder, 2011; Tomás et al., 2020). The central appeal of hope therapy lies in its structured, goal-oriented approach and broad applicability. Currently, hope therapy has been successfully implemented in education and clinical recovery contexts, demonstrating effectiveness in enhancing well-being, self-efficacy, and psychological resilience (Cheavens and Guter, 2018; Chen C. et al., 2024; Chen X. et al., 2024). These factors are positively associated with adaptive coping skills in school environments (Fenwick-Smith et al., 2018; Nowland and Qualter, 2020; Tomás et al., 2020). Gallagher et al. (2017) highlighted that hope therapy fosters coping strategies and perseverance in learning. Despite its academic benefits, such as improving test performance (Feldman and Kubota, 2015), research applying hope therapy to broader aspects of students’ school experiences—including their psychological well-being and school adaptation—remains relatively sparse.

This study integrates hope therapy with an eight-week mental health curriculum to enhance Chinese high school freshmen’s hope, pathways thinking, and motivational thinking, thereby improving their school adaptation systematically. It seeks to address two core research questions: (1) Can hope therapy be effectively incorporated into a mental health curriculum to create a scalable intervention model that helps students overcome academic, environmental, and interpersonal challenges during school transition? (2) What are the intervention effects and mechanistic roles of hope-therapy-based mental health curriculum in promoting school adaptation? Specifically, this study employs pre-tests to identify critical stabilization points for school adaptation, ensuring optimal timing for intervention. Using a pre-and post-test randomized controlled experimental design, matched groups were assigned to assess whether changes in students’ hope levels predict improvements in school adaptation. Furthermore, the study will evaluate the applicability and effectiveness of hope therapy within the Chinese educational context. The findings not only expand the theoretical foundation of hope therapy but also offer a scientific framework for designing mental health programs and practical intervention strategies to address school adaptation challenges among adolescents, providing both academic insights and educational relevance.

## 2 Methods

### 2.1 Participants

The sample for this study consisted of 892 adolescents (558 girls, 62.6%) who were assessed following their transition to high school (Grade 10). These participants were drawn from 16 classes in a mid-tier high school located in a county town in Guizhou Province, China.

### 2.2 Measures

#### 2.2.1 Hope

Hope was measured using the Trait Hope Scale (THS, Shi and Tian, 2009) based on Snyder’s Hope model. This scale comprises 8 self-report items measuring two dimensions of hope. The Cronbach’s *α* for the THS was initially 0.85. In the current sample, the Cronbach’s *α* for the THS was 0.91, indicating excellent internal consistency. Agency thinking was assessed through 4 items (e.g., “I am energetic and tirelessly pursue my goals,” Cronbach’s *α* = 0.77). Pathways thinking was evaluated using 4 items (e.g., “I can come up with many ways to overcome difficulties,” Cronbach’s *α* = 0.72). Responses were recorded on a 4-point Likert scale ranging from 1 (definitely false) to 4 (definitely true).

#### 2.2.2 School adaptation

In the pretest, school adaptation was measured using the Adolescence Psychological Adaptation Scale (APAS, Chen et al., 1995). This scale consists of 20 self-report items designed to assess four dimensions of adaptation, including physiological adaptation, learning adaptation, emotional adaptation, and interpersonal adaptation. Responses were recorded on a 5-point Likert scale ranging from 1 (strongly agree) to 5 (strongly disagree). The Cronbach’s *α* for the APAS was initially 0.65. In the current sample, the Cronbach’s *α* for the APAS was 0.66.

In the formal experiment, school adaptation was measured using the Scale of School Adaptation (SSA) for Junior High School Students (Hou, 2016). This scale consists of 27 self-report items assessing five dimensions of adaptation, with an initial Cronbach’s *α* of 0.96. In the current sample, the Cronbach’s α for the SSA was 0.89. School attitude was evaluated with 6 items (e.g., “I am satisfied with school life,” Cronbach’s *α* = 0.95). Peer relationships were measured using 6 items (e.g., “My classmates dislike me,” reverse-coded, Cronbach’s *α* = 0.89). Teacher-student relationships were assessed with 5 items (e.g., “I feel misunderstood by my teachers,” reverse-coded, Cronbach’s *α* = 0.93). Academic adaptation was measured using 5 items (e.g., “I complete my homework conscientiously,” Cronbach’s *α* = 0.90). Behavioral adaptation was evaluated through 5 items (e.g., “I often fail to follow classroom rules,” reverse-coded, Cronbach’s *α* = 0.88). Responses were rated on a 5-point Likert scale ranging from 1 (strongly disagree) to 5 (strongly agree).

### 2.3 Experimental design

**Figure 1:**
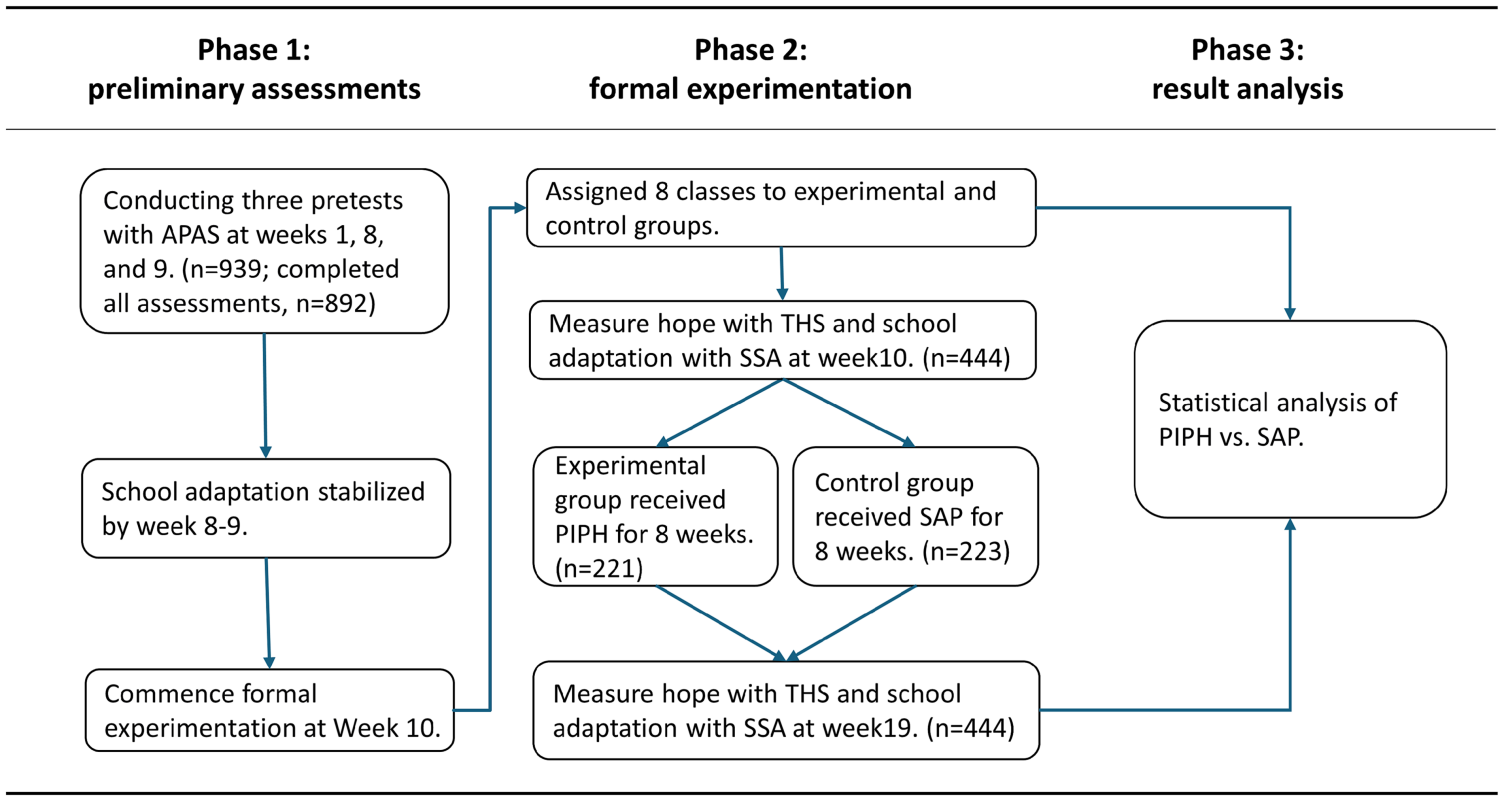
Research processes. APAS, Adolescence Psychological Adaptation Scale; THS, Trait Hope Scale; SSA, Scale of School Adaptation; PIPH, Psychological Intervention Program based on Hope therapy; SAP, Standard Adaptation Program.

Figure 1 illustrates the three main phases of this study.

#### 2.3.1 Phase 1: Pretest

**Figure 2:**
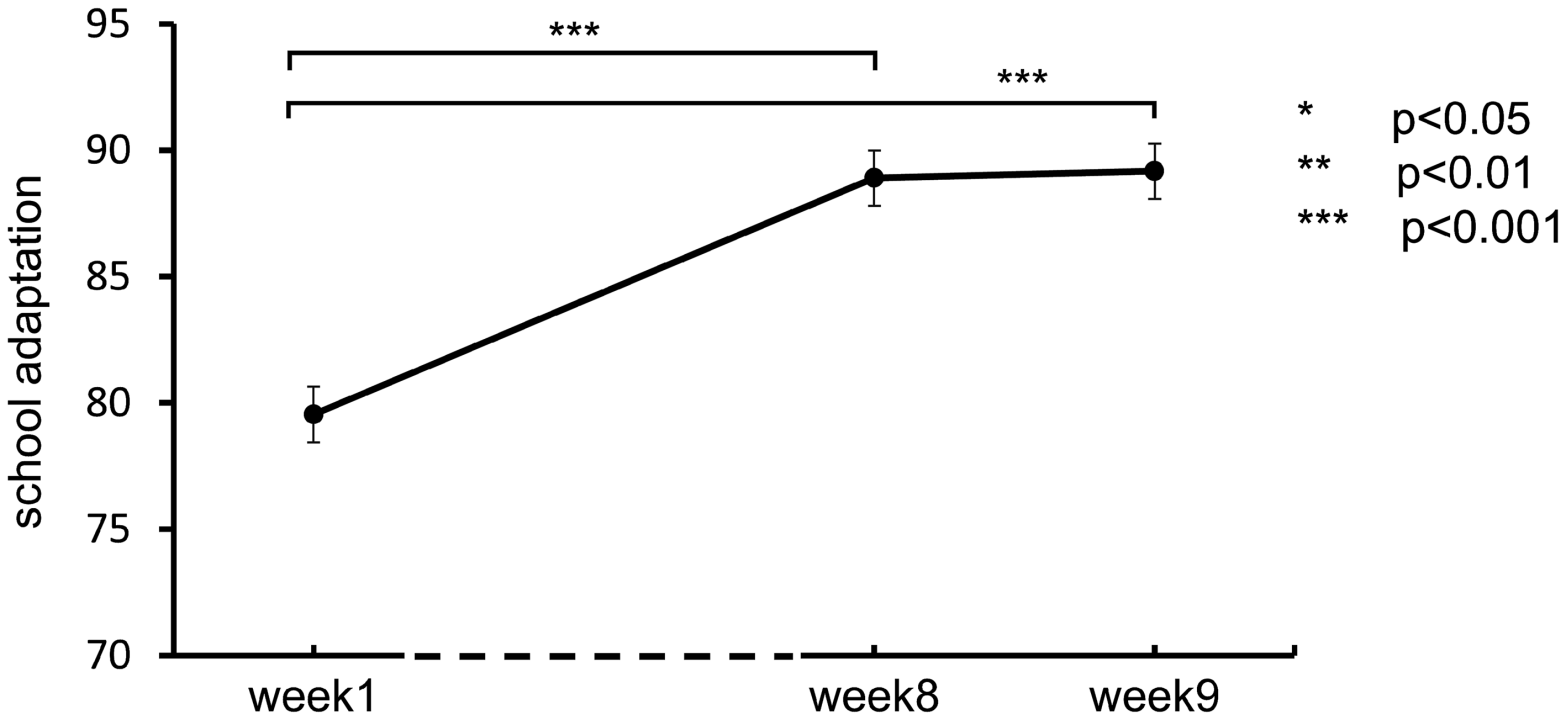
School adaptation development during week 1–9.

As students will gradually adapt to the school after enrollment, we decided to initiate intervention experiments after their school adaptation level is relatively stable, thus preventing overestimating the intervention effectiveness due to the natural dissipation of transitional maladjustment. So, three pretests were conducted with 939 freshmen at three time points during the first semester of Grade 10 (Week 1, Week 8, and Week 9). The APAS was administered to measure school adaptation. A total of 892 students completed all three assessments (completion rate = 95.0%). As shown in Figure 2, the school adaptation scores were as follows: Week 1, *M(SD)* = 79.54(16.38); Week 8, *M(SD)* = 88.90(16.03); Week 9, *M(SD)* = 89.17(16.41). Repeated measures ANOVA revealed a significant improvement in school adaptation between Weeks 1 and 8, with no significant change between Weeks 8 and 9, suggesting that school adaptation had stabilized. Based on these findings, the formal intervention began in Week 10, when adaptation was considered relatively stable.

#### 2.3.2 Phase 2: Intervention

A randomized controlled pretest-posttest design was employed, with a 2 (pretest vs. posttest) × 2 (experimental group vs. control group) structure. From the pretest participants, 444 students from 8 classes were selected through class cluster sampling to ensure representativeness based on gender, academic baseline, and school adaptation level. A *post hoc* power analysis indicated that our sample size of 444 participants provided more than sufficient statistical power (1-*β* = 0.99) to detect small to medium effect sizes (*d* = 0.59) at *α* = 0.05. To facilitate group-based intervention delivery, entire classes were randomly assigned to the experimental group (*n* = 221, 96 boys and 125 girls) or the control group (*n* = 223, 90 boys and 133 girls). The experimental group received an 8-week psychological intervention program based on hope therapy, while the control group participated in an 8-week standard adaptation program focusing on school adjustment, emotional regulation, and interpersonal skills. Changes in hope and school adaptation were assessed before and after the intervention using the Trait Hope Scale (THS) and the Scale of School Adaptation (SSA). We specifically employed different school adaptation scales across Phase 1 and Phase 2 to mitigate potential practice effects caused by repeated measurements of school adaptation. Phase 2 utilized the SSA with enhanced reliability, and validity, and construct validity aligned with the study’s theoretical framework. The pre-intervention school adaptation data from Phase 1 were solely employed to determine the optimal timing for intervention implementation and were not compared with the experimental data from Phase 2.

#### 2.3.3 Phase 3: Data analysis

Various statistical analyses were conducted to evaluate the effects of the hope therapy intervention relative to the standard adaptation program, providing insights into the practical impact of hope therapy.

### 2.4 Design of the hope-therapy-based intervention program

The psychological intervention program in this study is developed based on the mental health curriculum standards for Chinese high school students. This program is tailored to the characteristics of the Chinese education system, meets the psychological development needs of high school students, and is aligned with the school’s curriculum schedule. Each intervention session lasts 40 min, conducted once a week over 8 sessions (total duration: 6 h). The program is implemented as group counseling in class, aiming to foster collective growth through interactive guidance. Each session consists of three standard modules: an introduction (5 min) to engage students with the theme; a thematic activity (30 min) where key content is explored through interactive exercises; and a summary and after-class task module (5 min) to help students consolidate their learning and reflect on their experiences for further practice. All therapeutic activities were redesigned to reflect Chinese school scenarios, such as exam-related stressors and dormitory relationship management, while the course emphasized group-based experiential learning over individual counseling.

Following Snyder’s Hope Therapy and its’ school application advice (Pedrotti et al., 2008), the program incorporates the four core elements of Snyder’s Hope Theory: hope instilling, goal setting, agency thinking, and pathways thinking. The detailed hope-therapy-based contents of the 8 sessions are as follows:

Hope instilling: students were introduced to hope theory and its applications to school adaptation through activities designed to cultivate optimism and envision positive futures.

Goal setting: activities aimed to help students clarify their objectives for high school life and studies by guiding them to establish overarching goals and specific sub-goals. Students were encouraged to set reasonable goals—challenging yet attainable through effort.

Agency thinking: students learned strategies to maintain their drive toward achieving goals. These strategies included using positive self-talk, focusing on sleep, nutrition, and exercise to boost energy, leveraging past successes, visualizing future accomplishments, and incorporating self-reward mechanisms throughout the process.

Pathways thinking: students were instructed on methods for adapting and problem-solving. They learned to break goal paths into specific steps, list all feasible routes to achieve objectives, assess the pros and cons of each route, and evaluate potential difficulties along with solutions. Students also pre-planned their progress toward achieving goals. The specific course topics and design principles are summarized below:

### 2.5 Statistical analyses

All variables were found to conform to the assumption of normality, meeting the multivariate normality criteria outlined by Kline (2016). A range of statistical analyses was employed to examine the intervention effects and investigate the dynamic relationship between hope levels and school adaptation. First, Pearson correlation analysis was conducted to examine the relationships between hope, school adaptation and their sub-dimensions. Independent sample *t*-tests were used to compare baseline equivalence between the experimental and control groups prior to the intervention. Paired-sample *t*-tests and mixed multivariate analysis of variance (mixed MANOVA) were used to assess pre-to-post intervention changes in hope and school adaptation within the experimental group to determine intervention effects. Finally, cross-lagged structural equation modeling (SEM) was performed to investigate the dynamic relationships and causal mechanisms between hope and school adaptation. Statistical analyses were conducted using Mplus (version 8.3) and SPSS (version 27.0) (Table 1).

**Table 1:** The design of the hope-therapy-based intervention program.

Hope therapy content	Course title	Design approach
Hope Instilling	My Future Is Not a Dream	Activities like future visioning and storytelling were used to inspire hope.
	I Am Born to Excel	Introduced the multiple intelligence theory and the personal strength assessments.
Goal Setting	Pursuing Dreams	Interactive games and discussions encouraged translating dreams into goals.
	Setting Goals the SMART Way	Taught SMART principles for setting specific, measurable, achievable goals.
Agency Thinking	Overcoming Inferiority	Videos and discussions addressed self-doubt and built motivation.
	Building True Confidence	Group activities boosted self-esteem and reinforced positive self-evaluation.
Pathways Thinking	Finding Effective Study Methods	Case studies and group discussions promoted adaptive learning strategies.
	Exploring Career Knowledge	Introduced career planning concepts to reduce uncertainty and promote planning.

## 3 Results

### 3.1 Relationships between hope and school adaptation

To examine the relationships between hope and school adaptation, along with its sub-dimensions, Pearson correlation analyses were conducted using pretest data from the formal experiment. The results are summarized in Table 2. The findings indicate significant positive correlations between hope and overall school adaptation, as well as its specific dimensions (*r* = 0.16–0.50, *p* < 0.01). These results suggest that students with higher levels of hope exhibit better school adaptation across key domains. Furthermore, both components of hope—agency thinking and pathways thinking—were significantly associated with the dimensions of school adaptation. Notably, agency thinking demonstrated the strongest correlation with academic adaptation (*r* = 0.41, *p* < 0.001), highlighting that students with higher action-oriented thinking are more effective in addressing academic challenges.

**Table 2:** Correlations of latent factors of school adaptation and hope.

Variables	1	2	3	4	5	6	7	8	9
1. Hope	–								
2. Agency thinking	0.90^***^	–							
3. Pathways thinking	0.87^***^	0.57^***^	–						
4. School adaptation	0.35^***^	0.30^***^	0.32^***^	–					
5. School attitude	0.31^***^	0.30^***^	0.26^***^	0.86^***^	–				
6. Peer relationships	0.19^***^	0.11^*^	0.24^***^	0.79^***^	0.56^***^	–			
7. Teacher-student relationships	0.29^***^	0.25^***^	0.26^***^	0.80^***^	0.56^***^	0.57^***^	–		
8. Academic adaptation	0.44^***^	0.41^***^	0.37^***^	0.66^***^	0.54^***^	0.30^***^	0.48^***^	–	
9. Behavioral adaptation	0.14^**^	0.10^*^	0.16^***^	0.74^***^	0.56^***^	0.56^***^	0.53^***^	0.36^***^	–

^*^p < 0.05, ^**^p < 0.01, ^***^p < 0.001.

Grey shaded values represent total scores, while non-shaded values represent subscale scores.

### 3.2 Pretest differences between experimental and control groups

Table 3 shows the pre-test descriptive data of the experimental group and control group students, which serve as the starting point for each variable before the intervention experiment begins. All variables met normality thresholds prior to group assignment (|skewness| < 2, |kurtosis| < 7), with complete distribution characteristics including minimum/maximum values documented in Supplementary Table 1. An independent-sample *t*-test was performed to assess differences between the experimental and control groups at the pretest stage in terms of hope and school adaptation. As shown in Table 3, no significant differences were observed between the two groups across all variables (*t* ranging from −0.97 to 1.51, *p* > 0.05). These findings support the statistical equivalence of the groups prior to the intervention, providing a reliable baseline for subsequent comparisons.

**Table 3:** Pretest differences between experimental and control groups.

Variables	Experimental group (*n* = 221)		Control group (*n* = 223)		*t*-Test (*df* = 442)	
	*M*	*SD*	*M*	*SD*	*t*	*p*
Hope	10.36	1.93	10.34	1.93	0.08	0.937
Agency thinking	9.70	2.29	9.66	2.29	0.15	0.879
Pathways thinking	11.02	2.10	11.02	2.05	−0.02	0.982
School adaptation	3.74	0.61	3.74	0.57	−0.06	0.950
School attitude	3.60	0.81	3.64	0.76	−0.45	0.655
Peer relationships	4.05	0.79	4.13	0.75	−0.97	0.332
Teacher-student relationships	3.67	0.78	3.56	0.76	1.51	0.131
Academic adaptation	3.41	0.66	3.42	0.64	−0.10	0.920
Behavioral adaptation	4.01	0.74	4.00	0.72	0.15	0.883

Grey shaded values represent total scores, while non-shaded values represent subscale scores.

### 3.3 Comparative effects between experimental and control groups

Mixed MANOVA revealed significant interaction effects between time (pre- and post-intervention) and group (experimental and control group) for hope and school adaptation. Wilks’ Lambda = 0.91, *F*(1, 442) = 45.76, *p* < 0.001, partial *η^2^* = 0.09, suggesting a medium effect size; and for school adaptation, Wilks’ Lambda = 0.96, *F*(1, 442) = 11.00, *p* = 0.001, partial *η^2^* = 0.02, suggesting a small effect size. Time factors for all variables were significant. For hope, *F* = 72.77 to 156.28, *p* < 0.001, partial *η^2^* = 0.14 to 0.26, indicating a large effect size. For school adaptation, *F* = 4.58 to 16.73, *p* < 0.05, partial *η^2^* = 0.01 to 0.04, indicating that these effect sizes meet the threshold for minimal practical significance, with statistically significant effects and practical implications (detailed data available in Table 4).

**Table 4:** Results of mixed MANOVA for each variable across different time points and groups.

Variables	Time				Group			Interaction			
	Wilks’ Lambda	*F*	*p*	Partial *η*^2^	*F*	*p*	Partial *η*^2^	Wilks’ Lambda	*F*	*p*	Partial *η*^2^
HP	0.74	156.28^***^	<0.001	0.26	10.11^**^	0.002	0.02	0.91	45.76^***^	<0.001	0.09
AGT	0.78	125.71^***^	<0.001	0.22	10.19^**^	0.002	0.02	0.92	37.1^***^	<0.001	0.08
PWT	0.86	72.77^***^	<0.001	0.14	7.13^**^	0.008	0.02	0.96	21.09^***^	<0.001	0.05
SCA	0.96	16.73^***^	<0.001	0.04	2.42	0.120	0.01	0.98	11.00^***^	<0.001	0.02
SCT	0.98	7.55^**^	0.006	0.02	1.56	0.212	0.00	0.98	11.40^***^	<0.001	0.03
PRL	0.97	12.38^***^	<0.001	0.03	0.01	0.939	<0.01	0.99	4.13^*^	0.043	0.01
TSR	0.97	13.51^***^	<0.001	0.03	6.65^**^	0.010	0.02	0.99	3.52	0.061	0.01
ACA	0.98	8.15^**^	0.005	0.02	2.52	0.113	0.01	0.98	10.01^**^	0.002	0.02
BEA	0.99	4.58^*^	0.033	0.01	1.15	0.285	0.00	0.99	2.08	0.150	0.01

*p < 0.05, **p < 0.01, ***p < 0.001.

HP, hope; AGT, agency thinking; PWT, pathways thinking; SCA, school adaptation; SCT, school attitude; PRL, peer relationships; TSR, teacher student relationships; ACA, academic adaptation; BEA, behavior adaptation.

Grey shaded values represent total scores, while non-shaded values represent subscale scores.

For variables such as hope, agency thinking, pathways thinking, school adaptation, school attitude, peer relationships, and academic adaptation, significant interaction effects of time-group were observed. Subsequently, we conducted simple effects analyses for all variables. This was done not only to comprehensively explore the relationships among variables but also to maintain consistency in the analytical approach despite the minimal number of variables with non-significant interaction effects.

The results of the simple effects analysis showed that in the experimental group, there were significant differences over time (pre- and post-intervention) for all variables related to hope (*M* = 1.29 to 1.65, *p* < 0.001) and school adaptation (*M* = 0.13 to 0.22, *p* < 0.05). In the control group, there were significant differences over time for the hope variable (*M* = 0.39 to 0.49, *p* < 0.001), but no significant differences were found for the school adaptation variable (*M* = −0.02 to 0.06, *p* > 0.05). The experimental group experienced significantly greater gains in hope and its dimensions, as well as school adaptation (detailed data available in Table 5).

**Table 5:** Group*time pairwise comparisons.

Group	Variables	Pretest		Posttest		Test (post-pre)		
		*M*	*SE*	*M*	*SE*	*M*	*SE*	*t*
Experimental	Hope	10.36	0.13	11.83	0.13	1.47^***^	0.11	<0.001
	Agency thinking	9.70	0.15	11.35	0.15	1.65^***^	0.14	<0.001
	Pathways thinking	11.02	0.14	12.30	0.14	1.29^***^	0.14	<0.001
	School adaptation	3.74	0.04	3.93	0.04	0.19^***^	0.04	<0.001
	School attitude	3.60	0.05	3.82	0.06	0.22^***^	0.05	<0.001
	Peer relationships	4.05	0.05	4.26	0.05	0.21^***^	0.05	<0.001
	Teacher student relationships	3.67	0.05	3.86	0.06	0.19^***^	0.05	<0.001
	Academic adaptation	3.41	0.04	3.61	0.05	0.19^***^	0.05	<0.001
	Behavior adaptation	4.02	0.05	4.15	0.05	0.13*	0.05	0.012
Control	Hope	10.34	0.13	10.78	0.13	0.44^***^	0.11	<0.001
	Agency thinking	9.66	0.15	10.15	0.15	0.49^***^	0.14	<0.001
	Pathways thinking	11.02	0.14	11.41	0.13	0.39^**^	0.14	0.005
	School adaptation	3.75	0.04	3.77	0.04	0.02	0.04	0.583
	School attitude	3.64	0.05	3.61	0.06	−0.02	0.05	0.657
	Peer relationships	4.13	0.05	4.18	0.05	0.06	0.05	0.292
	Teacher student relationships	3.56	0.05	3.62	0.06	0.06	0.05	0.203
	Academic adaptation	3.42	0.04	3.41	0.05	−0.01	0.05	0.826
	Behavior adaptation	4.00	0.05	4.03	0.05	0.03	0.05	0.621

*p < 0.05, **p < 0.01, and ***p < 0.001.

Grey shaded values represent total scores, while non-shaded values represent subscale scores.

**Figure 3:**
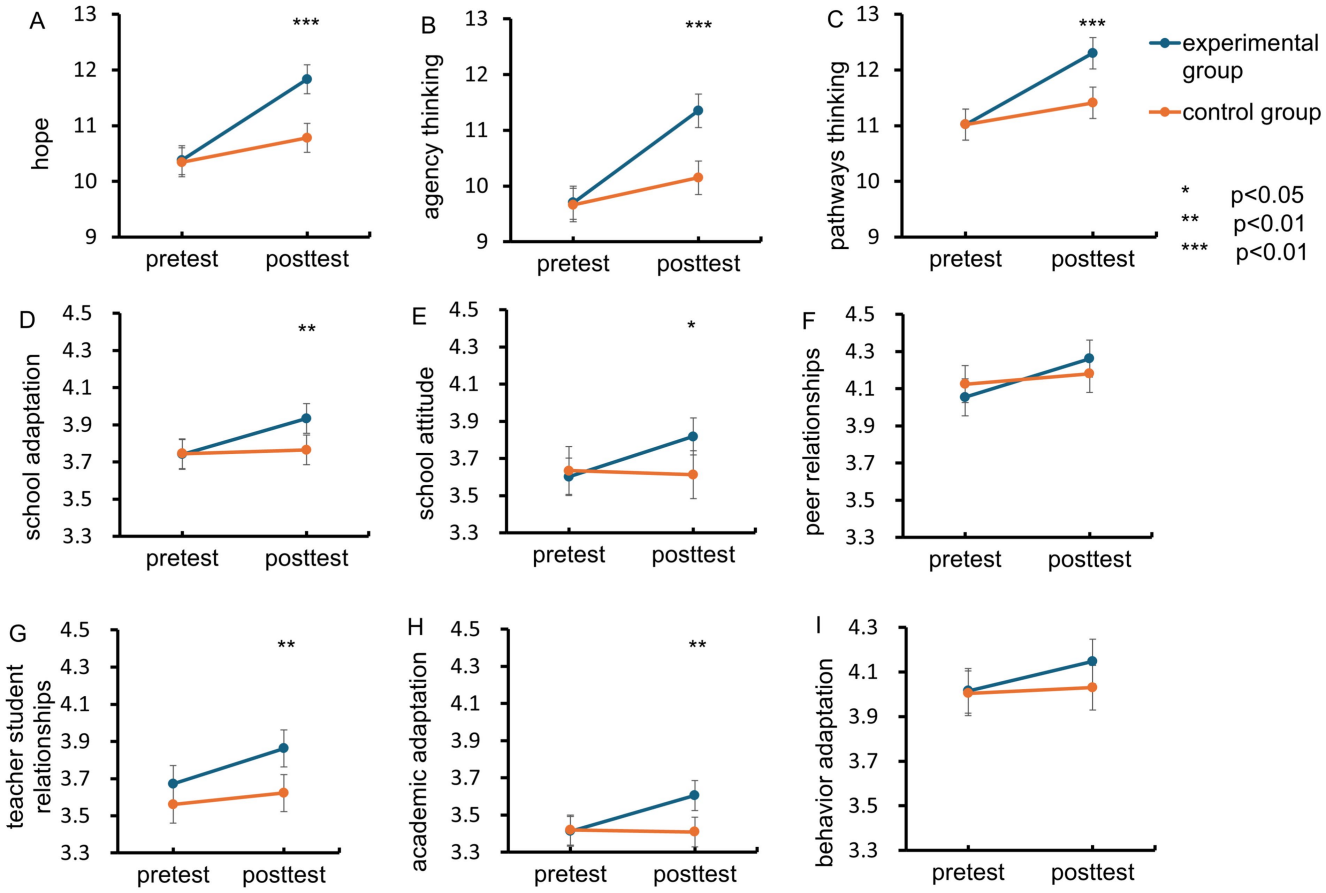
Individual data and averages for baseline and endpoint. **(A)** Hope, **(B)** agency thinking, **(C)** pathways thinking, **(D)** school adaptation, **(E)** school attitude, **(F)** peer relationships, **(G)** teacher-student relationships, **(H)** academic adaptation, **(I)** behavioral adaptation. Error bars are ± 2 SEM, The asterisks above the line chart indicate significant *p*-values based on independent-samples *t*-tests for the experimental group and the control group.

To provide a more intuitive and comprehensive understanding of these changes, and to further explore the overall impact of the intervention on various related aspects, we use graphical representations to vividly demonstrate them. Figure 3 illustrates the changes in hope and school adaptation levels between the experimental and control groups before and after the intervention. Notably, hope, agency thinking, and pathway thinking showed the largest improvements, with additional positive changes observed in academic adaptation and teacher-student relationships (*p* < 0.01). In contrast, the control group demonstrated smaller and less consistent improvements, with several results failing to reach statistical significance. These findings further validate the superior effectiveness of hope-therapy-based interventions relative to conventional mental health programs.

### 3.4 Intervention effects of hope-therapy-based mental health curriculum

A paired-sample *t*-test was conducted to evaluate the changes in hope and school adaptation levels within the experimental group before and after the intervention. As summarized in Table 6, the experimental group exhibited significant improvements across all variables (*p* < 0.001). Specifically, hope levels and its components increased by 1.29–1.65, *t*(220) = 8.78 to 12.66, with Cohen’s *d* ranging from 0.59 to 0.85 while school adaptation improved by 0.19, *t*(220) = 4.89. Cohen’s *d* = 0.33. According to Cohen’s (1988) benchmarks, hope and its dimensions showed moderate improvement (*d* = 0.59) after the intervention, while school adaptation manifested smaller but educationally meaningful gains (*d* = 0.33), surpassing the minimum important difference threshold (*d* > 0.20) (Lakens, 2013). The dimensions of school adaptation showed varying degrees of improvement, ranked as follows: school attitude, peer relationships, teacher-student relationships, academic adaptation, and behavioral adaptation. These results underscore the positive impact of hope-therapy-based interventions on enhancing students’ school adaptation.

**Table 6:** Intervention effects within the experimental group.

Variables	Pretest		Posttest		Paired differences			*t*-test (*df* = 220)	
	*M*	*SD*	*M*	*SD*	*M*	*SD*	*d*	*t*	*p*
Hope	10.36	1.93	11.83	1.99	1.47	1.72	0.85	12.66^***^	<0.001
Agency thinking	9.70	2.29	11.35	2.29	1.65	2.18	0.76	11.25^***^	<0.001
Pathways thinking	11.02	2.10	12.30	2.07	1.29	2.18	0.59	8.78^***^	<0.001
School adaptation	3.74	0.61	3.93	0.69	0.19	0.59	0.33	4.89^***^	<0.001
School attitude	3.60	0.81	3.82	0.90	0.22	0.79	0.27	4.06^***^	<0.001
Peer relationships	4.05	0.79	4.26	0.78	0.21	0.81	0.26	3.81^***^	<0.001
Teacher-student relationships	3.67	0.78	3.86	0.83	0.19	0.77	0.25	3.69^***^	<0.001
Academic adaptation	3.41	0.66	3.60	0.80	0.19	0.72	0.27	3.97^***^	<0.001
Behavioral adaptation	4.01	0.74	4.15	0.73	0.13	0.78	0.17	2.52^*^	0.012

^*^p < 0.05 and ^***^p < 0.001.

Grey shaded values represent total scores, while non-shaded values represent subscale scores.

### 3.5 Cross-lagged analysis of hope and school adaptation

We conducted confirmatory factor analysis (CFA) on the THS following the steps outlined by Anderson and Gerbing (1988) to assess its construct validity. The results indicated good model fit: *χ^2^* = 127.11, *df* = 19, *p* < 0.001, CFI = 0.94, TLI = 0.91, GFI = 0.97, RMR = 0.026, SRMR = 0.041, RMSEA = 0.079, indicating that the THS demonstrates strong construct validity. The confirmatory factor analysis also supported the hypothesized factor structure of the SSA, with a good model fit (*χ^2^* = 47.47, *df* = 5, *p* < 0.001, GFI = 0.96, CFI = 0.95, TLI = 0.90, RMR = 0.022, SRMR = 0.044, RMSEA = 0.138), indicating that the SSA demonstrates acceptable construct validity.

**Figure 4:**
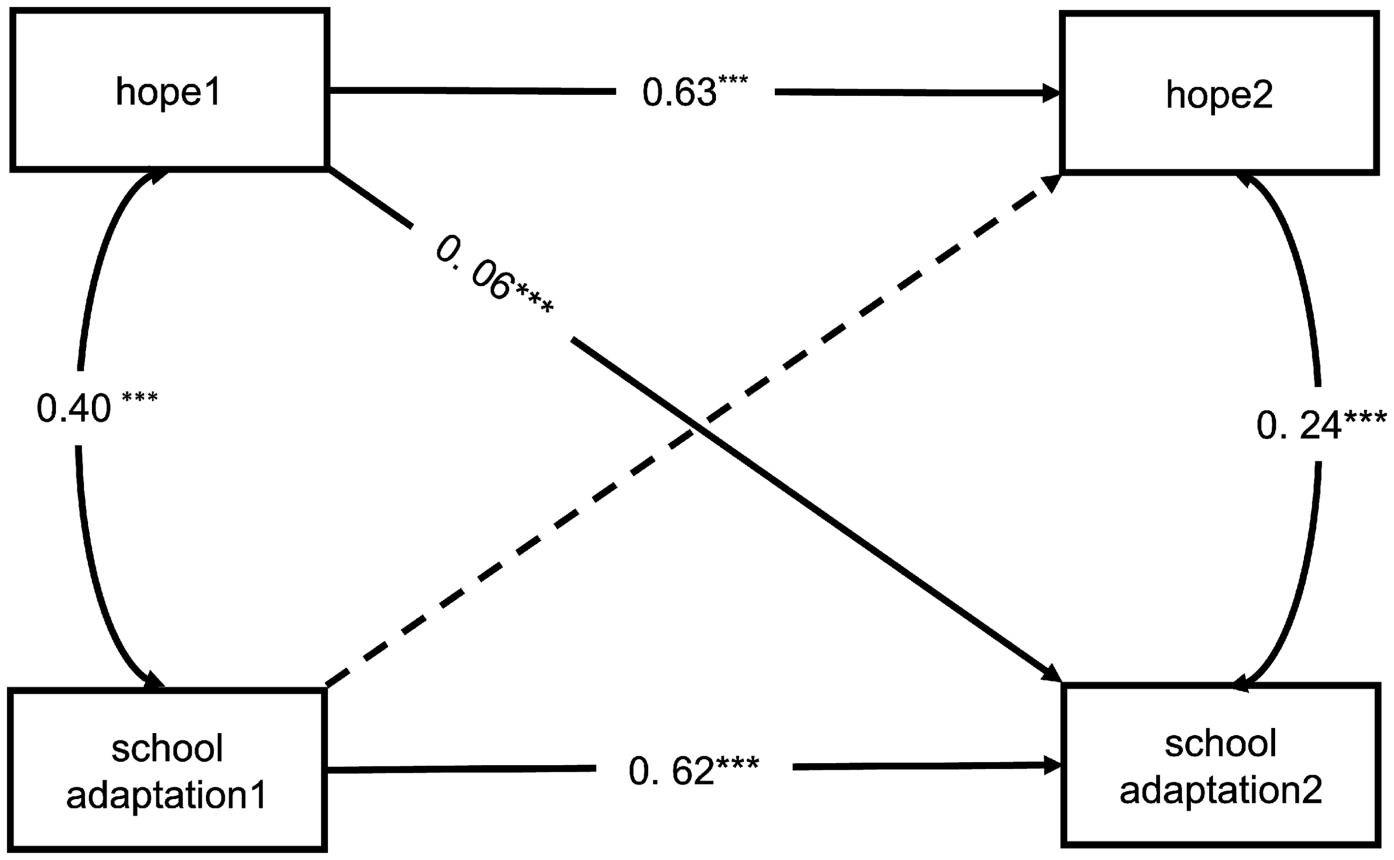
Cross-lagged reciprocal effects models between hope and school adaptation. Baseline measurements (hope1 and school adaptation1) were obtained at Week 10 prior to intervention initiation, with post-intervention assessments (hope2 and school adaptation2) conducted at Week 19. *p < 0.05, **p < 0.01, ***p < 0.001. Significant pathways are shown in solid lines, and insignificant pathway is shown in dashed line.

To better understand the causal direction and effects of the intervention program, we used a bidirectional cross-lagged model to examine the reciprocal predictive relationships between changes in hope and school adaptation. The model demonstrated a good fit with the data (*χ^2^* = 41.62, *df* = 6, *p* < 0.001; CFI = 0.92; TLI = 0.90, GFI = 0.93, SRMR = 0.056, RMSEA = 0.071). As Figure 4, baseline hope levels significantly predicted subsequent school adaptation improvements (*β* = 0.064, *p* < 0.001), whereas the reverse effect of school adaptation on hope was not significant (*β* = 0.227, *p* = 0.089). These results empirically demonstrate that hope can significantly foster school adaptation, and the reverse causality is ruled out.

## 4 Discussion

This study aimed to address the prevalent school adaptation challenges faced by Chinese high school freshmen by integrating hope therapy into psychological education. Responding to the critical need for scalable mental health interventions, this study developed and validated a structured, eight-week hope-therapy-based intervention curriculum, and evaluated the intervention’s effectiveness using a pretest-posttest control group experimental design. The results revealed significant improvements in hope and school adaptation among students in the experimental group, with their performance surpassing those of their pre-intervention level and those of the control group, highlighting the positive intervention effects of hope on school adaptation. Moreover, the longitudinal cross – lagged modeling provides effective causal evidence. Specifically, the hope level measured at week 10 before the intervention (hope 1) significantly predicted the improvement of school adaptation at week 19 after the intervention (hope 2). This indicates that hope can significantly foster school adaptation. These findings collectively support the effects and mechanism of hope-based interventions on strengthening school adaptation. Therefore, this study provides an innovative and alternative model for school education, with a strong focus on raising youths’ hope to develop meaningful lives (Lillard, 2023).

This study designed and validated the hope-therapy-based intervention curriculum. Our study provides step-by-step procedures and empirical evidence on the effectiveness of integrating hope therapy into an 8-week structured program within regular school settings, which showed satisfying scalability and integration within the regular school framework to address prior limitations of highly targeted and resource-intensive interventions (Stormshak et al., 2011; Tornivuori et al., 2023). The curriculum incorporated core hope therapy principles, including goal setting, pathway thinking, and agency thinking, which were systematically integrated into each session. The significant improvements in both hope levels and school adaptation in the experimental group, compared to the control group receiving regular adaptation lessons, demonstrate the added value of hope therapy within an existing curriculum framework. The intervention was delivered by trained school teachers within a natural classroom setting, without reliance on external specialists. This highlights the potential for widespread adoption in various educational contexts with appropriate teacher training and resource allocation. The curriculum aligns with Chinese educational policies promoting mental health education, making it a solid basis for broader empirical implementation and policy alignment. Based on these findings, authorities responsible for mental health curricula development could integrate structured hope-building modules into existing curriculum frameworks, utilizing this study’s validated 8-week intervention model. Educational policymakers could support pilot programs implementing hope-based mental health curricula in high schools, especially in regions with limited psychological resources. Furthermore, teacher training programs could incorporate hope therapy techniques to enhance educators’ capacity to deliver evidence-based interventions. These steps would operationalize China’s mental health promotion policies while addressing adolescents’ multidimensional adaptation needs through scalable, school-embedded approaches, supporting broader empirical implementation and policy alignment.

Hope therapy was particularly suitable for addressing school adaptation challenges due to its emphasis on fostering positive expectations, goal-setting, adaptive coping strategies, emotional regulations, and self-efficacy, which align closely with the developmental needs of adolescents navigating academic and social transitions. Unlike traditional interventions that primarily target maladaptive behaviors or symptom reduction, hope therapy adopts a strength-based, future-oriented perspective (Snyder, 2011). By focusing on goal-directed thinking and enhancing students’ sense of agency and pathways to achieve their goals, the intervention equips adolescents with proactive strategies to manage transitions effectively. This distinctive approach not only addresses immediate adaptation challenges but also builds long-term psychological resources. This is consistent with existing intervention studies, which suggest that positive psychological interventions have a better effect on improving high school students’ psychological resources (e.g., promoting emotions, coping styles, resilience, and self-efficacy) than traditional psychological interventions (Lei et al., 2021). This reinforces the importance of targeting positive emotions and cognitive-motivational processes in mental health interventions for long-term impact, as supported by the broaden-and-build theory (Fredrickson, 2001). These results offer practical guidelines for mental health educators. For instance, the integration of hope, pathways thinking, and agency thinking into standard classroom practices could foster long-term resilience and adaptability among students. Schools and educators can adopt this model to provide preventional and systematic mental health support to large student populations in transitional periods over reactive approaches (Nazari et al., 2024).

This study represents a pioneering effort to integrate hope therapy into a curriculum-based intervention, demonstrating its feasibility within a Chinese cultural context. While hope therapy originates from Western individualistic traditions that emphasize personal agency and goal-setting (Snyder, 2011), this study shifts the focus from intervention effectiveness of individual well-being using White, English-speaking populations to school adaptation in Chinese student groups. Although our intervention did not utilize explicit cultural adaptation frameworks, we employed systematic localization in content and pedagogy. Given the emphasis on academic performance and examination pressures in Chinese schools (Chen C. et al., 2024; Chen X. et al., 2024), this study highlights the potential for positive psychology approaches to mitigate stress and enhance adaptation in such high-pressure environments. All therapeutic activities were redesigned to reflect Chinese school scenarios, such as exam-related stressors and dormitory relationship management, while the course emphasized group-based experiential learning over individual counseling. Our classroom activities leveraged shared academic struggles (e.g., school transitions and Gaokao preparation) as communal hope-building anchors. This aligns with cross-cultural studies showing hope is a promising intervention direction for groups facing stressors, including but not limited to academic success (Snyder et al., 2002), substance abuse (Assari et al., 2024), and learning disorders (Ben-Naim et al., 2017). These design choices ensured that the intervention focused on hope’s transcultural cognitive components—agency and pathways thinking—which have demonstrated cross-cultural predictive validity for academic adaptation.

This study demonstrates methodological and practical innovations that strengthen its contributions to school psychology and mental health research. First, it identifies the natural stabilization time for school adaptation, enabling precise intervention timing to evaluate the intervention effectiveness. This methodological advancement addresses a common limitation in prior studies where intervention effects could be confounded by natural adjustment over time. Second, the pretest-posttest control group experimental design and the cross-lagged modeling approach provide deeper insights into the directional relationship between hope and school adaptation, offering a stronger empirical basis for causal interpretations. This methodological rigor enhances the credibility and generalizability of the findings. Third, the research provides compelling evidence of mental health as a key determinant of academic success and overall well-being. By demonstrating that psychological interventions can yield tangible improvements in school adaptation, it reinforces the importance of integrating mental health curricula into educational policies and practices.

While the study establishes a strong foundation, several limitations should be acknowledged. First, the sample was confined to a single county-level high school in China. Although this setting allowed for controlled implementation, the cultural specificity and homogeneous socioeconomic background of participants may limit the generalizability of findings to urban educational contexts or other cultural environments. Second, the 6-month experimental time frame, while sufficient for assessing short-term intervention effects, precludes conclusions about the long-term sustainability of observed outcomes. The developmental trajectory of school adaptation throughout high school remains to be explored. Future research can explore several directions to build upon these findings. First, longitudinal studies tracking long-term effects of the intervention would provide insights into its sustainability and potential for fostering resilience throughout high school. Second, examining the intervention’s impact on schools and students across diverse cultural and socio-economic contexts could enhance its generalizability and inform culturally responsive adaptations. Second, longitudinal studies tracking participants’ adaptation trajectories over multiple academic years would clarify the durability of intervention effects. Cross-cultural comparative studies involving diverse educational systems (e.g., urban vs. rural, Eastern vs. Western contexts) could enhance the ecological validity of the intervention model, providing insights into its sustainability and potential for improving school adaptation throughout high school. Furthermore, integrating mixed-methods designs with qualitative interviews would yield a richer understanding of students’ subjective experiences and the contextual factors mediating intervention effectiveness. Additionally, incorporating qualitative assessments could also provide richer data on students’ lived experiences, further informing program development.

## 5 Conclusion

This study demonstrates the significant potential of hope therapy in enhancing school adaptation for Chinese high school freshmen. By integrating hope therapy into an eight-week mental health curriculum, we provide a scalable, evidence-based intervention that addresses the psychological and social challenges students face during their critical transition to high school. The results indicate that hope therapy effectively increases students’ hope levels and improves various dimensions of school adaptation. Our longitudinal cross-lagged modeling further confirms that changes in hope predict improvements in school adaptation, underscoring the importance of fostering cognitive-motivational processes such as agency and pathways thinking.

In conclusion, this study highlights the transformative potential of hope therapy in fostering school adaptation among adolescents, offering a promising avenue for addressing the mental health needs of students in high-pressure educational systems. This approach aligns with contemporary trends in adolescent mental health research, advocating for preventive and early intervention strategies to enhance well-being and academic success. Despite its strengths, the study acknowledges certain limitations, including the sample’s cultural homogeneity and the short-term nature of the intervention. Future research should explore the long-term sustainability of these effects and test the intervention in diverse educational settings to further validate and refine the model. Additionally, cross-cultural studies could provide deeper insights into the universal applicability of hope therapy across different educational and socio-cultural contexts.

## Data Availability

The raw data supporting the conclusions of this article will be made available by the authors, without undue reservation.
