# Supplementary material for: Hope therapy brings hope: an empirical study of a curriculum intervention to enhance school adaptation of Chinese high school freshmen
Source: Front Psychol. 2025 Apr 28;16:1555364. doi: 10.3389/fpsyg.2025.1555364 (PMC12066303; doi:10.3389/fpsyg.2025.1555364)
Supplement: Supplementary file 1 [file Table_1.docx]

**Appendix.**

**Supplementary Table 1.** Pre-split full summary statistics of the pretest (n=444).

| Variables | Mean | *SD* | skewness | kurtosis | min | max |
| --- | --- | --- | --- | --- | --- | --- |
| hope | 10.35 | 1.97 | -0.17 | 0.69 | 4.00 | 16.00 |
| agency thinking | 9.68 | 2.17 | 0.11 | 0.49 | 4.00 | 16.00 |
| pathways thinking | 11.02 | 2.12 | -0.18 | 0.28 | 4.00 | 16.00 |
| school adaptation | 3.74 | 0.50 | 0.03 | -0.53 | 2.44 | 5.00 |
| school attitude | 3.62 | 0.66 | -0.01 | -0.61 | 2.14 | 5.00 |
| peer relationships | 4.09 | 0.53 | -0.38 | -0.36 | 2.67 | 5.00 |
| teacher-student relationships | 3.61 | 0.73 | -0.11 | -0.73 | 2.00 | 5.00 |
| academic adaptation | 3.42 | 0.65 | -0.07 | -0.17 | 1.60 | 5.00 |
| behavioral adaptation | 4.01 | 0.56 | -0.06 | -0.67 | 2.75 | 5.00 |
